# A mixed-methods pilot study of domiciliary nasal high-flow therapy for breathlessness in people with chronic obstructive pulmonary disease who do not qualify for domiciliary long-term oxygen therapy

**DOI:** 10.1177/17534666251314722

**Published:** 2025-03-12

**Authors:** Natasha Smallwood, Amy Pascoe, Catherine Buchan, Aaron K. Wong, David Currow, Brian Le

**Affiliations:** Department of Respiratory and Sleep Medicine, Alfred Health, Prahran VIC, Australia; Respiratory Research@ The Alfred, School of Translational Medicine, Monash University, Level 6, Alfred Centre, 99 Commerical Rd, Prahran, VIC 3004, Australia; Respiratory Research@ The Alfred, School of Translational Medicine, Monash University, Prahran VIC, Australia; Respiratory Research@ The Alfred, School of Translational Medicine, Monash University, Prahran VIC, Australia; Palliative Medicine, Peter MacCallum Cancer Centre, Parkville, VIC, Australia; Palliative Medicine, Royal Melbourne Hospital, Parkville, VIC, Australia; Faculty of Science, Medicine and Health, University of Wollongong, Wollongong, NSW, Australia; Faculty of Medicine and Health Sciences, University of Melbourne, Parkville, VIC, Australia; Palliative Medicine, Peter MacCallum Cancer Centre, Parkville, VIC, Australia; Palliative Medicine, Royal Melbourne Hospital, Parkville, VIC, Australia

**Keywords:** breathlessness, chronic obstructive pulmonary disease, nasal high-flow therapy

## Abstract

**Background:**

High-flow nasal oxygen (HFNO) therapy delivers humidified, heated air with flow rates of up to 60 L/min with oxygen entrained. HFNO has advantages over conventional oxygen therapy, including precise and reliable fraction of inspired oxygen delivery, therefore is recommended as first-line treatment for people with acute hypoxaemic respiratory failure.

**Objectives:**

This pilot study aimed to determine the feasibility and acceptability of domiciliary nasal high flow (NHF) without entrained oxygen for people with chronic obstructive pulmonary disease (COPD) and severe breathlessness.

**Design:**

Single-arm, mixed-methods, pilot study of an 8-day, air-only NHF intervention in adults with COPD and severe breathlessness not requiring domiciliary oxygen therapy.

**Methods:**

Participants were educated and advised to use NHF for ⩾7 h per night for 7 nights with day use as desired. Patient-reported outcome measures were assessed on Days 3, 5 and 8. Primary outcome: feasibility. Secondary outcomes: breathlessness (dyspnoea), fatigue, quality of life, physical function, sleep, tolerability and safety. Acceptability was also assessed through semi-structured interviews.

**Results:**

Fifteen participants were enrolled (mean age 73.6; 40% women; mean FEV_1_ 41% predicted, mean DLCO 43.0% predicted; mean modified Medical Research Council score 3.7). Thirteen (87%) completed the trial, with 8 (54%) keeping the device at the end of the trial and 3 (20%) continuing use long-term. Adherence varied, with average daily usage higher amongst participants who kept the device compared to those who returned it (6.8 h ± 2.3 h vs 3.4 h ± 3.7 h). No changes in worst breathlessness (mean = 0.7, SD = 1.2, *p* = 0.109), dyspnoea mastery (mean = 0.3, SD = 0.6, *p* = 0.176) or fatigue (mean = 0.0, SD = 2.4, *p* = 1.00) were observed at Day 8 compared to baseline. No significant adverse events were reported. Qualitative interviews demonstrated subjective improvements in breathlessness, dry mouth and sputum production for some participants, whilst others found NHF uncomfortable. Fear of NHF dependence and concerns regarding long-term running costs were reported.

**Conclusion:**

Domiciliary NHF was a feasible intervention, albeit with varied adoption and acceptability. These trial implementation outcomes may have affected preliminary effectiveness outcomes. Further research is required to determine what role domiciliary NHF may have for people with COPD and severe breathlessness.

**Trial registration:**

ACTRN12621000044820.

## Introduction

Chronic obstructive pulmonary disease (COPD) is an incurable illness characterised by chronic respiratory symptoms that cause persistent and often progressive airflow obstruction.^1^ Globally there are estimated to be 384 million COPD cases.^2,3^ In Australia, 30% of people aged over 75 years have COPD; it is the fifth leading cause of death and the third leading cause of avoidable admission.^4–7^

Breathlessness describes *‘a subjective experience of breathing discomfort that consists of qualitatively distinct sensations that vary in intensity*^8^*’* that affects up to 98% of people with severe COPD^9,10^ and is the primary cause for 14% of all emergency ambulance callouts.^11^ People with breathlessness often also suffer from fatigue, anxiety, decreased function and reduced quality of life.^12,13^ Importantly, breathlessness is a predictor of unscheduled healthcare use, hospitalisation and mortality in people with chronic lung diseases.^14,15^ Whilst improved treatments for COPD have prolonged survival,^16^ symptom management remains poor.^17–19^

Treating severe chronic breathlessness is challenging due to the advanced nature of the underlying diseases that contribute to this symptom. Domiciliary, long-term oxygen therapy (LTOT) improves survival in people with COPD with severe resting hypoxaemia (PaO_2_ <55 mmHg),^20^ however, has no role in people with only moderate hypoxaemia (PaO_2_ >60 mmHg) with severe chronic breathlessness.^21,22^ In that group, a randomised controlled trial found that air (given via a sham concentrator) was as effective as oxygen therapy in alleviating breathlessness,^23^ suggesting that air movement on the face may be helpful for symptom relief. Current low-flow oxygen therapy delivery systems may deliver insufficient flow for symptom relief and importantly lack airflow heating and humidification, which can lead to desiccation of the airway mucosa and patient discomfort.^24^

The myAirvo 2™ is a nasal high-flow therapy (NHF) device that delivers heated, humidified air with or without entrained oxygen, with patients able to tolerate high flows of up to 60 L/min.^25^ NHF washes out carbon dioxide from airway dead space in the lungs to improve ventilation and generates a low-level positive airway pressure (4–5 cmH_2_O) that may reduce the work of breathing.^26,27^ NHF entrained with oxygen is an established treatment in acute care settings, shown to improve respiratory rate and oxygenation for people with acute respiratory failure,^28^ and provide symptom relief for people with cancer with severe breathlessness.^29,30^

Long-term, domiciliary NHF (flows of 20 L/min for 7 h/day) together with LTOT has been demonstrated to be both safe and superior to LTOT alone in people with severe COPD and hypoxaemia.^31^ Effects include reduced admissions, and improved ventilation, exercise capacity and breathlessness, with no adverse events.^31^ Further, a recent study of NHF was shown to alleviate experimentally induced dyspnoea in healthy volunteers independently of oxygen entrainment.^32^ Domiciliary NHF with air-only has great potential, yet has not been studied in people with severe COPD and breathlessness with only moderate hypoxaemia. This pilot study aimed to determine the feasibility and acceptability of domiciliary NHF for the treatment of severe chronic breathlessness in people with COPD who do not qualify for domiciliary oxygen.

## Materials and methods

### Study design and setting

A single-arm, open-label, mixed-methods, pilot study of NHF was conducted over fourteen months with primary aims of feasibility in line with the *Medical Research Council framework for development and evaluation of complex interventions.*^33^ Participants were recruited from inpatient and outpatient tertiary respiratory care and palliative care clinics based at Alfred Health and Royal Melbourne Hospital in Melbourne, Australia. The reporting of this study conforms to the CONSORT guidelines.^34^

### Participants

Adults with physician-confirmed, medically optimised, severe COPD (including severe emphysema without co-existing airflow obstruction, as confirmed by pulmonary function tests within the last 24 months with FER ⩽70% and FEV_1_ ⩽50%, OR radiological evidence of emphysema on CT chest scan within the last 5 years and DLCO ⩽50% within the last 24 months) with moderate to severe breathlessness as defined by a modified Medical Research Council score ⩾3, who were not using domiciliary LTOT via a concentrator for 16 h/day (oxygen saturations >92% at rest) were eligible to participate. People who used exertional oxygen therapy from portable oxygen cylinders for exertion-induced hypoxaemia were eligible.

Exclusion criteria included: acute exacerbations of COPD in the past 4 weeks (i.e. unstable disease), enrolment in other clinical trials that may impact breathlessness, commencement of opioids or benzodiazepines within last 14 days or anticipated commencement whilst on study, previous adverse reaction to NHF, clinician predicted survival <7 days, pregnant or breastfeeding, chronic alcoholism or drug abuse, untreated pneumothorax and contraindications to NHF use (including requiring non-invasive or invasive ventilation; altered level of consciousness (Glasgow Coma Scale 10 or below); suspected or known base of skull fracture; inability to maintain own airway or unable to tolerate nasal prongs; recent surgery in last 30 days to the nose or upper; significant facial fractures or trauma in last 30 days; nasal obstruction or nasal packing; and current SARS-CoV-2 (COVID-19) infection in accordance with local COVID-19 infection control procedures at the time of the study which prohibited the use of aerosol-generating procedures including NHF).

### Sample size

Given the primary outcome of feasibility, no formal sample size was calculated for this pilot study.

### Intervention and data collection

An 8-day trial of NHF using air-only (no entrained oxygen) via the myAirvo 2™ device (Fisher and Paykel Healthcare, New Zealand Australian Registry of Therapeutic Goods entry 282584). All trial procedures were completed either in the hospital or at home by trained study staff. Following baseline assessment, a respiratory nurse or respiratory scientist who had received training to use myAirvo 2™ conducted the initial setup of NHF.

A standard guide for NHF titration (Table 1) was developed and individualised as needed. Over a maximum of 100 min, settings were up-titrated to the target flow of 30 L/min at 37°C, or to the highest setting below this level that the patient comfortably tolerated.

**Table 1.** Nasal high-flow therapy titration.

Time (min)	Flow rate (L/min)	Temperature (°C)
0	10	31
10	15	34
20	20	37
30	25	37
40	30	37
*50*	*35*	*37*
*60*	*40*	*37*
*70*	*45*	*37*
*80*	*50*	*37*
*90*	*55*	*37*
*100*	*60*	*37*

Participants were given the option of temporarily experiencing a higher flow rate (as indicated in italics) during the titration period before returning to the target flow rate of 30 L/min.

Participants were educated on how to use NHF at home and advised to wear it for a minimum of 7 h per night for 7 days, with additional day use as desired. Patient-reported outcome measures were assessed on Days 3 and 5 and immediately post-intervention.

Participants were given the option to retain the myAirvo 2™ device on indefinite loan at no cost at the completion of the trial. Participants were asked to participate in a semi-structured interview to explore their experiences and acceptance of NHF.

### Outcomes

The primary outcome was feasibility and acceptability as measured by enrolment of 15 participants over 6 months with ⩾80% of enrolled participants progressing to study completion. Acceptability was also assessed through semi-structured interviews with participants at the completion of the study. Interviews were conducted by phone or teleconferencing by a trained member of the research team who was not involved in the intervention delivery.

Secondary outcomes included: best, worst, average breathlessness and average fatigue over last 24 h at 9 am and 4 pm daily by 0–10 Numerical Rating Scale (NRS); quality of life (Chronic Respiratory Disease Questionnaire Self-Administered Standardized (CRQ-SAS^35^); physical function (Resource Utilisation Groups – Activities of Daily Living (RUG-ADL) score^36^ and the Australia-modified Karnofsky Performance Scale (AKPS^37^); sleep (Pittsburgh Sleep Quality Index (PSQI^38^) and validated wrist-based sleep tracker (watchPAT ONE)); tolerability (study retention and adherence with NHF using automatically recorded actual device usage); safety (serious adverse events using Common Terminology Criteria for Adverse Events version 5.0 (CTCAE v5.0) assessed at all study contacts); rescue medications (i.e. opioids/anxiolytic/bronchodilator on inpatient drug charts or medication diary); and daily steps (FitBit Charge 2 wearable fitness tracker).

### Statistical analyses

Demographic and other baseline data are reported descriptively. Categorical data are presented as frequencies and percentages unless stated otherwise. For continuous data, mean and standard deviation or median and interquartile range are presented as summary tables unless stated otherwise. Morning and evening symptom scores were combined to create average daily scores. Changes from baseline in secondary measures at Day 8 were analysed using a paired *t*-test with alpha = 0.05 and reported using mean difference with a 95% confidence interval.

Semi-structured interviews were recorded and transcribed verbatim with all identifying data removed. Transcripts were coded and synthesised thematically according to the principles of iterative thematic analysis as outlined by Braun and Clarke.^39^

## Results

### Recruitment and participant demographics

**Figure 1.**
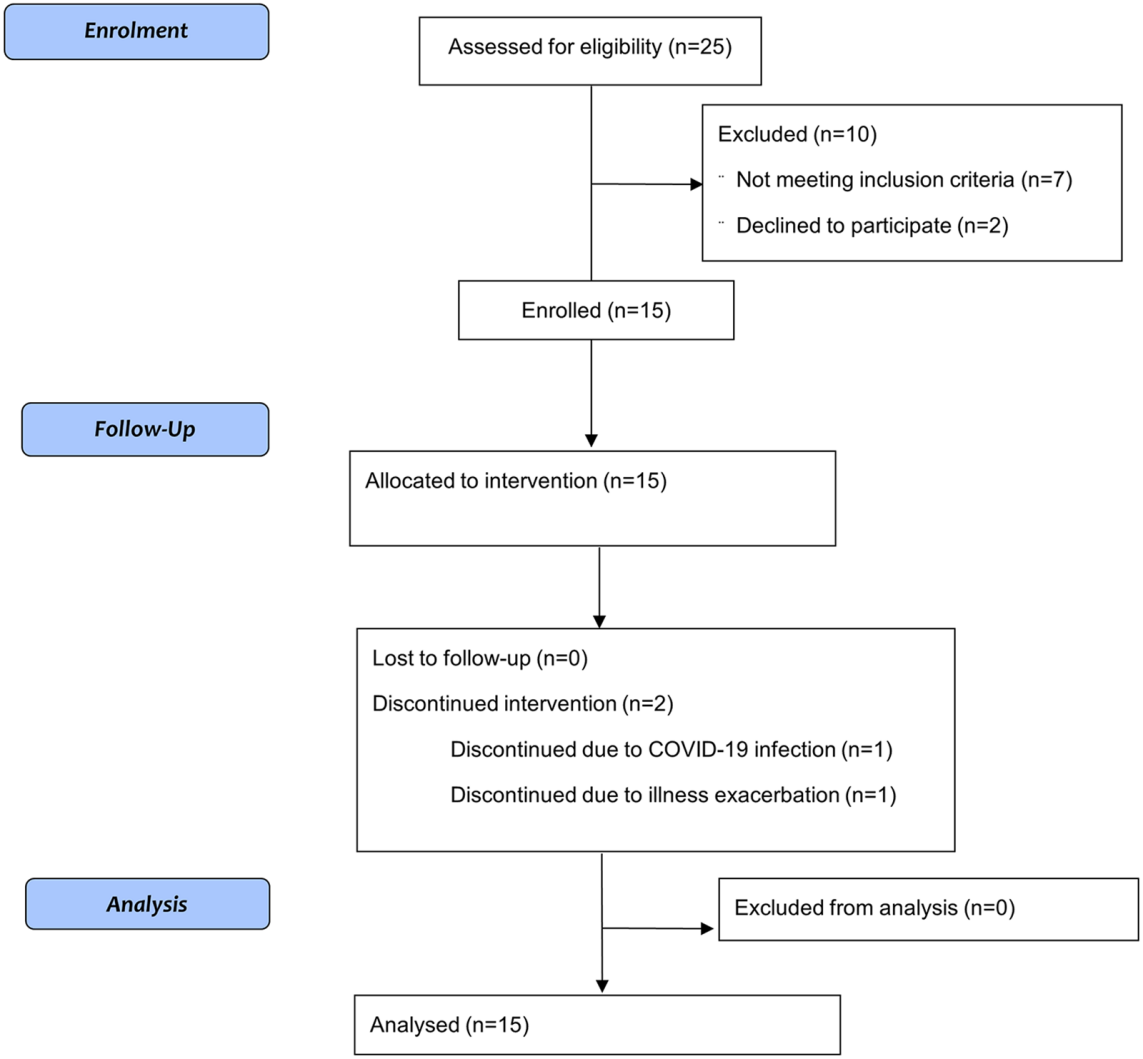
CONSORT study flow diagram.

Fifteen participants were enrolled over a period of 9 months (September 2021 and April to December 2022). Trial activities were suspended for 6 months (October 2021 to March 2022) due to local COVID-19–related restrictions regarding the usage of aerosol-generating procedures; one participant was enrolled prior to the suspension and the remaining 14 were enrolled within an eight-month period after the trial activities were resumed. Three participants had preserved spirometry but reduced gas transfer (DLCO ⩽50%) with severe emphysema on CT scan. Thirteen participants (86.6%) completed the study, with two discontinuing due to illness unrelated to the intervention (Figure 1). Participants had a mean age of 73.6 years (SD = 6.7) and 40% (*n* = 6) were female (Table 2). Day 1 and Day 8 visits were conducted in person, with the majority of participants (*n* = 11, 73.3%) receiving in-home visits for all trial procedures.

**Table 2.** Participant demographics (*n* = 15).

Characteristic	Mean	SD
Age (years)	73.6	6.7
	*n*	%
Gender		
Male	9	60.0
Female	6	40.0
Mental health co-morbidities		
Anxiety	6	40.0
Depression	3	20.0
mMRC score		
3	5	33.3
4	10	66.7
Intervention setting		
Inpatient	1	6.7
Outpatient	14	93.3
Availability of carer		
Lives with carer	6	40.0
Lives alone with carer available	3	20.0
Lives with non-carer	1	6.7
Lives alone, no carer available	5	33.3
	Mean	SD
Pulmonary function results		
Recency (months prior to baseline)	8.7	5.9
	Median	IQR
FEV_1_ absolute (L)	0.94	0.68–1.15
FEV_1_ predicted percentage (%)	41.0	22.00–41.00
FVC absolute (L)	2.72	2.23–3.33
FVC predicted percentage (%)	87.0	73.00–101.00
DLCO absolute (mL/min/mmHg) (*n* = 13)	8.89	6.79–12.00
DLCO predicted percentage (%) (*n* = 13)	43.0	36.00–54.00
	Mean	SD
Baseline sleep study (*n* = 7)		
Recorded sleep time	483.4 min [8 h 3 min]	62.9 min [1 h 3 min]
% REM	21.6	4.4
% Oxygen saturation ⩽88%	14.7	22.1
pAHI	19.7	15.9
	*n*	%
OSA category		
Mild	4	57.1
Moderate	2	28.6
Severe	1	14.3
Using home portable oxygen therapy for exertional hypoxemia	4	26.7
Prior completion of pulmonary rehabilitation	10	66.7
	Median	IQR
Emergency presentations in past 24 months due to respiratory illness (*n* = 13)		
Not resulting in admission	0	0.0–0.0
Resulting in admission	0.0	0.0–2.0

OSA category cut-offs: none = pAHI <5; mild = pAHI 5 ⩽ 15; moderate = pAHI 15 ⩽ 30 severe = pAHI >30.

mMRC, modified Medical Research Council; FEV_1_, forced expiratory volume in 1 second; FVC, forced vital capacity; DLCO, diffusing cpacity for carbon monoxide; REM, rapid eye movement sleep; OSA, obstructive sleep apnoea; pAHI, PAT-derived apnoea-hypopnea index; PAT, peripheral arterial tonometry; REM, rapid eye movement.

### NHF usage and acceptability

Average daily device time used was obtained from 10 of 13 participants who completed the intervention; the remainder did not return the device in a timely manner to enable accurate measurement of total usage time. Average daily use time was 5 h 25 min (SD 3 h 13 min), with only four participants (40%) meeting the suggested average daily usage of >7 h. The median recorded flow rate was 30 L/min (IQR 30–30) and the median recorded temperature was 37°C (IQR 34–37).

Eight participants (53.3%) elected to keep the myAirvo 2™ device on indefinite loan at the end of the trial period with the stated intention to continue use; however, five of these participants returned the device within 8 months of study completion. The remaining seven participants, including the two participants withdrawn due to unrelated illnesses, returned the device, generally citing difficulty or discomfort in using the device, or concerns about dependence on the device. Average daily usage time trended higher amongst participants who initially kept the device (6 h 45 min ± 2 h 16 min) compared to those who returned it (3 h 23 min ± 3 h 40 min).

### Patient-reported outcomes

Mean changes in all other secondary outcomes are reported in Table 3. A statistically significant increase in average breathlessness was detected at the end of the trial period. No other significant changes were detected.

**Table 3.** Repeated measures.

Scale	Timepoint				Change from baseline	
	Baseline (day −2 to day 1)	Day 3	Day 5	Day 8	Day 8 – Baseline Mean difference [95% CI]	*p*
Breathlessness NRS	*n* = 13	*n* = 9	*n* = 9	*n* = 9	*n* = 9	
Worst	6.3 (1.8)	6.1 (2.3)	7.2 (1.5)	6.5 (2.0)	0.72 [−0.20, 1.65]	0.109
Best	2.8 (1.7)	3.2 (2.5)	4.1 (2.6)	3.9 (2.7)	0.56 [−0.54, 1.65]	0.276
Average	4.1 (1.4)	4.6 (2.2)	4.8 (2.0)	5.2 (2.1)	1.11 [0.14, 2.09]	0.030
Fatigue NRS	*n* = 13	*n* = 9	*n* = 9	*n* = 9	*n* = 9	
Average	5.6 (2.4)	5.2 (2.4)	5.7 (2.2)	5.8 (2.9)	0.00 [-1.87, 1.87]	1.00
	*n* = 7	*n* = 4	*n* = 5	*n* = 4	*n* = 3	
PSQI	10.3 (2.8)	11.0 (2.6)	10.8 (1.9)	10.3 (1.5)	0.33 [−1.10, 1.77]	0.423
CRQ-SAS	*n* = 14	*n* = 8	*n* = 8	*n* = 11	*n* = 11	
Dyspnoea	3.1 (1.3)	3.5 (1.3)	4.0 (0.5)	3.3 (1.0)	0.03 [−0.74, 0.80]	0.929
Fatigue	3.0 (1.3)	2.8 (1.2)	3.4 (1.2)	3.1 (1.4)	−0.07 [−0.25, 0.12]	0.432
Emotional function	4.0 (1.2)	4.0 (0.8)	4.2 (1.0)	4.1 (0.9)	−0.17 [−0.57, 0.23]	0.369
Mastery	3.1 (0.8)	3.4 (0.6)	3.7 (0.8)	3.5 (1.0)	0.25 [−0.13, 0.63]	0.176
	*n* = 12	*n* = 11	*n* = 11	*n* = 10^a^		
Daily steps	1256.8 (668.1)	1551.6 (1163.9)	1283.7 (868.6)	744.0 (493.3)	−320.6 [−721.31, 80.11]	0.104
	*n* = 15			*n* = 12	*n* = 12	
AKPS	60.0 (12.5)	–	–	60.0 (13.5)	−0.83 [−2.67, 1.00]	0.339
RUG-ADL	*n* = 15	*n* = 13	*n* = 12	*n* = 12	*n* = 12	
RUG-ADL total	4.9 (1.9)	4.5 (1.9)	4.6 (2.0)	4.6 (2.0)	−0.17 [−0.53, 0.20]	0.339
Bed mobility	1.4 (0.8)	1.2 (0.6)	1.2 (0.6)	1.2 (0.6)	−0.17 [−0.53, 0.20]	0.339
Toileting	1.3 (0.7)	1.2 (0.6)	1.2 (0.6)	1.2 (0.6)	0.00 [–, –]	–
Transfer	1.1 (0.5)	1.2 (0.6)	1.2 (0.6)	1.2 (0.6)	0.00 [–, –]	–
Eating	1.1 (0.2)	1.1 (0.3)	1.1 (0.3)	1.1 (0.3)	0.00 [–, –]	–

Data are reported as means with SD in parentheses.

NRS scores reported as average of morning and evening scores.

a FitBit recording on final day does not include full 24 h.

AKPS, Australian Karnofsky performance score (higher score indicates higher functional status); CRQ-SAS, Chronic Respiratory Disease Questionnaire Self-Administered Standardized (higher score indicates higher quality of life); NRS, numerical rating score (higher score indicates worse symptom burden); PSQI, Pittsburg sleep quality index (higher score indicates worse sleep quality); RUG-ADL, resource utilisation group activities of daily living (higher score indicates more assistance required).

Five participants used rescue medications during the intervention, including Salbutamol (3 participants, mean frequency of use = 3 occasions), hydromorphone (1 participant, 2 occasions) and oral morphine solution (1 inpatient participant, 20 occasions).

Due to COVID-19–related restrictions, the majority of participants were unable to complete 6MWT assessments. In addition, eight participants were unable to complete watchPAT ONE sleep monitoring due to technical difficulties. Notably, all seven participants who completed watchPAT ONE sleep monitoring at baseline had measurements consistent with obstructive sleep apnoea (OSA) despite not having a prior formal or self-reported diagnosis.

### Safety and adverse events

No serious or unexpected adverse events were reported (Table 4). Reported adverse events included a burning sensation in the nose, chest pain and epistaxis, all of which were mild (CTCAE v5.0, grade 1) and resolved within 1 day. Of note, several participants described discomfort and other issues related to the usage of the device, including dry airways and throat pain, during the semi-structured interviews at the end of the trial period, which were not reported when prompted during the intervention.

**Table 4.** Adverse events.

Adverse event description	Grade CTCAE 5	Frequency	Related to NHF	Outcome
Burning sensation in nose	NA	1	Possible	Resolved in <1 day
Headache	1	1	Unlikely	Resolved in <1 day
Chest pain	1	1	Unlikely	Resolved in <1 day
Epistaxis	1	1	Possible	Resolved in <1 day
COVID-19 infection	NA	1	Unrelated	Discontinued intervention due to infection risk
Exacerbation of COPD		1	Unrelated	Hospital admission and discontinued intervention

COPD, chronic obstructive pulmonary disease; CTCAE, Common Terminology Criteria for Adverse Events; NHF, nasal high flow.

### Patient experiences and perceptions

Of thirteen participants who completed the trial, 10 completed end-of-trial semi-structured interviews. Major themes included perceived changes to symptoms and experiences with logistics regarding both the device and the trial itself (Table 5).

**Table 5.** Patient experiences and perceptions.

Themes	Subthemes	Participant quotes
Changes to COPD symptoms	Improvements in symptoms	*‘It’s just a bit easier to get breath.’ – P1* *‘It’s definitely helped. I don’t get such a dry mouth as I used to. . . I’m finding it good.’ – P2* *‘It seems to help a little bit, you know? I think I, there’s less phlegm in my chest.’ – P3*
	Discomfort from heat and humidification of equipment	*‘Yeah just the hot air was making it hard for me to sleep cos it was uncomfortable. And then when I woke up in the morning, I realized why I could hardly talk, my throat was so sore. . .and it made me cough a lot more.’ – P4* *‘And I, I did say this to [respiratory nurse], how you would go with that warm tubing. . . um, because it is quite warm, right? Um, in, in a, like a hot summer night. So [respiratory nurse] said to me, well, you just wouldn’t use it.’ – P1* *‘I just felt having something on my face all the time at night was not very comfortable.’ – P5*
	Unknown changes due to short-term nature of clinical trial	*‘It hasn’t changed much. . .after I’ve had the machine for a longer period, a month or so . . .we’re probably a little bit quick giving you the answer, not for the amount of time that I’ve had the machine’ – P6*
Inconveniences of the machine	Initial setup	*‘It was very, oh, well, the setup was brilliant. They explained it well. Well, um, I didn’t have any problems about setting it up when I got home.’ – P2* *‘It seemed simple to set up. I just couldn’t get it to work. . .I couldn’t get to the next stage.’ – P4*
	Varied sensitivity to the device noise	*‘I was very impressed with the silence of the machine. . .it was well-engineered’ – P8* *‘Yeah, at first I thought, oh, you know, how am I going to sleep with this noise. . . uh, but now it’s okay I’m used to it.’ – P8* *‘I think they need a bigger water tank.’ – P8*
	Dissatisfaction with the water chamber	*‘The only trouble I do have is getting the machine out because my, my hands aren’t strong enough or big enough to bring the water chamber out. Um, that’s the only problem I have.’ – P2* *‘Only gives you enough water for six hours. Well, I sleep eight, nine hours. So it’s sort of. . . was a bit of a hassle having to set the alarm at 11 o’clock. And then of course by the time you get up, go to the toilet. . .wait for the machine to warm up and everything else, you’re wide awake so then you can’t go back to sleep.’ – P9*
	Inconvenient placement of the device on the floor	*‘Um, yeah, and, and more like that tubing, I didn’t know where to put it, you know, like it kept, I think because the unit’s on the floor, and if I moved, it kind of dragged the tubing, dragged the things out of your nose . . .’ – P1* *‘No, apart from the chamber, you know, trying to get the, the water chamber out, that’s my only problem and I, I’ve got it low down. I’ve got it sitting on the floor.’ – P2*
	Varied use of the machine during the day/night	*‘But then, you know, everything’s difficult to do when it’s night-time [laughs]. I’m not sure how you could improve that actually. You know, so most people, you know, stumble around and find your glasses and put the lights on and you know, try and find the relevant dials and you know, but I dunno how you could improve on that.’ – P5* *‘During the day. I don’t like using it at night. . .I don’t, don’t like it, you know, very much. See, I prefer. . .I inhale through my nose, and exhale through my mouth. But if I sleep. . .I have my mouth open all the time. . .And I get a very dry mouth.’ – P3*
Long-term use of the machine	Concerns about dependency	*‘I don’t want to be sort of left high and dry when it goes. And, uh, um, oh, I want something to latch onto, I don’t want something to latch onto. It’s a matter of weaning myself off.’ – P8* *‘I’m really getting used to it and what worries me now is how am I going to get on without it,’ – P8* *‘I mean, I’m so positive about it and you know, oh God, why am I getting so positive? Because I’m, you know, going to have to learn to live without it.’ – P8*
	Ongoing costs of consumables and electricity	*‘No, not really. No, if it’s helping you, I think that’d be a small price to pay.’ – P1* *‘You know, the electricity, if it’s sky high. I guess I’d say right. I can’t afford it.’ – P2* *‘Yeah, the cost it’d be pretty heavy cause I think they told me you have to replace the breathing tube um monthly. . .I think they would cost a lot of money.’ – P8*

COPD, chronic obstructive pulmonary disease.

### Perceived changes to symptoms

Some participants reported subjective improvements in breathlessness, dry mouth and sputum production whilst in the study. Several participants described a sense of hope regarding NHF. By contrast, one person found the device suffocating and anxiety-inducing which led to discontinuation. Some participants found the heat and humidification to be uncomfortable with concerns about usage in hot weather. Some reported interruptions to sleep related to the equipment and heat generated. It was difficult for some participants to determine whether NHF was beneficial given the short duration of the study.

### Inconveniences of the machine

Although some participants described the machine as quiet, others reported sleep disruption and required gradual familiarisation. Despite the provision of adequate support by healthcare staff during initial setup, some expressed dissatisfaction or difficulties with the equipment, particularly regarding the refilling of the water chamber and the inconvenient placement of the device on the floor. One participant proposed a larger water tank. Despite advice to use the device at night, some participants preferred to use the machine during the day.

### Long-term use of the machine

Some participants expressed concerns about becoming dependent on the device. Ongoing costs of consumables and electricity contributed to some participants’ reluctance to use the machine long-term.

## Discussion

This pilot study of domiciliary NHF (without entrained oxygen) recruited and enrolled 15 participants with 13 participants (86.6%) completing the trial. Although the formal target of 15 participants enrolled within 6 months was not met, this was largely due to significant pandemic disruptions to research activities.

The observed statistically significant deterioration in average breathlessness scores should be interpreted cautiously given the broad 95% confidence interval which includes the mean change being below the clinically important difference of 1 point^40^ and small sample size in this pilot study which increases the risk of a false-positive observation.^41^ For this reason, we do not believe this represents a clinically significant change in breathlessness; future trials adequately powered to detect changes in this outcome are necessary. Other secondary outcomes assessing impacts on symptoms and quality of life showed no significant changes and semi-structured interviews revealed considerable variation in perceived benefit.

Participant adherence with recommended device usage of 7 h per night varied but was overall low. The proportion of participants who discontinued or heavily reduced their usage of the device within the first days of the trial indicates that initial setup, education and acclimatisation is a crucial period and may be a barrier to tolerable and beneficial NHF. This is consistent with prior studies of adherence to domiciliary respiratory supports. Continuous positive airway pressure (CPAP) therapy for people with OSA is a useful comparison, as both devices present similar user experiences and barriers to adherence. Early adherence to CPAP has been shown to be a key predictor of improved long-term adherence.^42^ Up to 15% of people with OSA decline CPAP usage after one night and as many as 50% will discontinue usage within 1 year.^43^ Similarly, LTOT adherence amongst people with COPD has been reported between 45 and 70%.^44^ Although the current study included one-on-one setup and support from a dedicated experienced respiratory nurse, which was positively received, it was evident that a longer supported acclimatisation period would be beneficial. Of note, seven participants in the current study had sleep monitoring data indicative of OSA, despite having no formal diagnosis. The presence of detectable OSA, diagnosed or otherwise, may be a confounder in future effectiveness studies and should be confirmed by polysomnogram and controlled for during recruitment or data analysis.

Some participants reported discomfort when using the device, including dry or burning sensations in their nose and throat, which may have been improved with adjustments to flow settings. Although all participants were prompted to report adverse events at regular intervals throughout the study, these events were not formally reported and only arose during the semi-structured interviews with a non-clinician researcher. This may reflect the tendency of some patients to under-report symptoms to their clinical care team.^45^ Some reported discomforts, including noise and the sensation of the device on the face during sleep, are inherent to the device which simply may not suit all people. Additionally, some participants found the device logistically cumbersome to operate in-home which may be at least partially alleviated with the provision of more suitable accessories and other updates to the design in future models. These findings are consistent with evidence from studies of long-term non-invasive ventilation usage in COPD, which indicate that the treatment burden of such devices can be high and many participants discontinue due to adverse events.^46,47^ Notably, some participants reported that the myAirvo 2™ device was preferable in terms of portability and stigma to their experiences or perceptions of LTOT. Gradual acclimatisation to wearing the device was a commonly reported theme which reinforces the importance of the early intervention period. For future trials, a longer intervention period with more scheduled inputs for technical and clinical support early in the intervention period may enhance uptake, adherence and importantly, participant comfort.

The current study utilised a number of digital monitoring technologies. Given this patient population generally has limited familiarity with digital technologies, the initial setup and education session necessarily included education on the usage of other trackers which may have diluted the focus on NHF. Difficulties utilising trackers contributed to incomplete data in the current study. Future trials of effectiveness may benefit from reduced or staggered introduction of digital trial devices to reduce the risk of overloading participants with information.

The current study was not sufficiently powered to detect differences in symptom outcomes and perceived effects reported in semi-structured interviews were mixed. Whilst some felt that NHF produced modest improvements, including changes to perceived functional capacity and breathlessness, others described no perceivable improvements. Some participants reported that the intervention period was too brief to determine whether the NHF was beneficial. In future, a longer intervention period may help to overcome these barriers and provide more robust information on clinical effectiveness. The number of participants who elected to keep the myAirvo 2™ device at the conclusion of the trial is, however, indicative that this therapy was of perceived value to half of the participants.

It should also be noted that at least one participant returned the device despite reporting minor benefits, citing concerns about the financial burden of ongoing consumables which were provided free of charge but only for 6 months post-trial. At the time of writing, the estimated monthly cost of consumables is approximately $AUD48 per month (estimate provided by Fisher & Paykel Healthcare). Financial burden of treatment costs is additive and can be burdensome for many people with chronic disease who are often older, under- or unemployed or otherwise reliant on aged care or disability pensions and socioeconomically disadvantaged.^7,48,49^

### Strengths and limitations

To our knowledge, this is the first study examining domiciliary, air-only NHF for people with COPD and severe breathlessness who do not qualify for domiciliary LTOT. The primary outcome of this study was significantly impacted by restrictions related to the COVID-19 pandemic. Given the feasibility aims of this pilot study, no sample size was calculated and the study is not adequately powered to detect differences in the exploratory secondary outcomes. Secondary outcomes are presented in that light and should not be over-interpreted. Overall adherence to therapy was lower than desired, which may have impacted perceived effects on symptoms. Given the current study advises participants to utilise NHF at night, future studies would benefit from collection and reporting of specific co-morbidities that may contribute to breathlessness, including heart failure and pulmonary hypertension. Future studies would benefit from a longer trial period, with more frequent scheduled supports, to enable participants to become more familiar with and acclimatised to the NHF device and trial procedures.

## Conclusions

This pilot study of air-only NHF in people with COPD with breathlessness who do not qualify for domiciliary oxygen indicates that a phase III trial is feasible, albeit refinement of the delivery would be beneficial. Preliminary data are not sufficient to determine the effectiveness of the intervention on symptom burden. Qualitative data from participants highlight barriers to usage including discomfort, logistical difficulties and concerns about long-term dependence, but also indicates that some participants perceive benefit from the therapy and elect to continue usage post-trial.

## Supplemental Material

sj-doc-1-tar-10.1177_17534666251314722 – Supplemental material for A mixed-methods pilot study of domiciliary nasal high-flow therapy for breathlessness in people with chronic obstructive pulmonary disease who do not qualify for domiciliary long-term oxygen therapy Supplemental material, sj-doc-1-tar-10.1177_17534666251314722 for A mixed-methods pilot study of domiciliary nasal high-flow therapy for breathlessness in people with chronic obstructive pulmonary disease who do not qualify for domiciliary long-term oxygen therapy by Natasha Smallwood, Amy Pascoe, Catherine Buchan, Aaron K. Wong, David Currow and Brian Le in Therapeutic Advances in Respiratory Disease

## Declarations

**Ethics approval and consent to participate:** This trial was sponsored by the Royal Melbourne Hospital. All trial activities were approved by, and conducted in accordance with, The Royal Melbourne Hospital Human Research Ethics Committee (HREC 2020/124). Governance approvals were additionally obtained from all trial sites (Alfred Health 430/21 and Peter MacCallum Cancer Centre 20/221 (no participants were enrolled at Peter MacCallum Cancer Centre)). Written consent was obtained from all trial participants. Trial details were prospectively registered on the Australian and New Zealand Clinical Trials Register (ANZCTR 12621000044820).

**Consent for publication:** All participants provided written consent for publication.

**Availability of data and materials:** The datasets used and/or analysed during the current study are available from the corresponding author on reasonable request.
